# Faith Cure

**Published:** 1895-06

**Authors:** 


A Dexter (Mich.) woman got so much faith in faith cures that she threw away her false teeth, expecting her natural teeth to grow in again. She waited six months, and now has neither faith nor teeth.

